# Examining the Effectiveness of Electronic Patient-Reported Outcomes in People With Cancer: Systematic Review and Meta-Analysis

**DOI:** 10.2196/49089

**Published:** 2024-07-31

**Authors:** Melissa Betty Perry, Sally Taylor, Binish Khatoon, Amy Vercell, Corinne Faivre-Finn, Galina Velikova, Antonia Marsden, Calvin Heal, Janelle Yorke

**Affiliations:** 1 Christie Patient Centred Research The Christie NHS Foundation Trust Manchester United Kingdom; 2 Division of Nursing Midwifery and Social Work School of Health Sciences, Faculty of Biology, Medicine & Health The University of Manchester Manchester United Kingdom; 3 Division of Cancer Science The University of Manchester Manchester United Kingdom; 4 Clinical Oncology Department The Christie NHS Foundation Trust Manchester United Kingdom; 5 Leeds Institute of Medical Research University of Leeds Leeds United Kingdom; 6 St James’s Institute of Oncology St James’s University Hospital Leeds United Kingdom; 7 Division of Population Health Health Services Research & Primary Care The University of Manchester Manchester United Kingdom; 8 School of Nursing The Hong Kong Polytechnic University Kowloon China (Hong Kong)

**Keywords:** telemedicine, patient-reported outcome measure, neoplasms, quality of life, systematic review, meta-analysis, randomized controlled trial

## Abstract

**Background:**

Electronic patient-reported outcomes (ePROs) are commonly used in oncology clinical practice and have shown benefits for patients and health resource use.

**Objective:**

The aim of this study was to compare the isolated effect of administering ePROs to patients with cancer versus a control condition.

**Methods:**

The PRISMA (Preferred Reporting Items for Systematic Reviews and Meta-Analyses) guidelines were followed. Randomized controlled trials evaluating ePRO interventions that aimed to improve health-related outcomes among patients with cancer were included. The primary outcome was health-related quality of life (HRQOL), and the secondary outcomes were symptoms, hospital admissions, unplanned visits, chemotherapy completion, survival, and satisfaction with care. The effect sizes of ePROs on health-related outcomes were analyzed as standardized mean differences (SMDs) with 95% CIs using a random effects model.

**Results:**

The search identified 10,965 papers, of which 19 (0.17%) from 15 studies were included. The meta-analysis showed an improvement in HRQOL at 3 months, measured by the Functional Assessment of Cancer Therapy–General (SMD 0.29, 95% CI 0.19 to 0.39), and at 6 months, assessed using various HRQOL measures (SMD 0.21, 95% CI 0.11 to 0.30). Of the 15 studies, 9 (60%) reported a positive signal on HRQOL, with two-thirds of the studies (n=6, 67%) including tailored patient advice and two-thirds (n=6, 67%) using clinician alert systems.

**Conclusions:**

The meta-analysis showed an improvement in HRQOL at 6 months and in Functional Assessment of Cancer Therapy–General scores at 3 months for studies that included tailored advice and clinician alerts, suggesting that these elements may improve ePRO effectiveness. The findings will provide guidance for future use and help health care professionals choose the most suitable ePRO features for their patients.

**Trial Registration:**

PROSPERO CRD42020175007; https://tinyurl.com/5cwmy3j6

## Introduction

### Background

A patient-reported outcome (PRO) has been defined as “a measurement of any aspect of a patient’s health status that comes directly from the patient” [1]. PROs can be more sensitive and reliable than outcomes reported by clinicians, particularly when reporting adverse events [2,3]. Electronic PROs (ePROs) refer to an electronic administration of the PROs. ePRO systems can present summary reports of patients’ health-related quality of life (HRQOL) and symptoms to the clinician in real time to enhance communication [4] and improve patient management and outcomes.

Many different types of ePRO systems have been developed to monitor and manage diseases, treatments, and symptoms [5] in a variety of patient groups. Guidelines published by the European Society for Medical Oncology recommend using ePROs for symptom monitoring in routine oncology care during systemic cancer treatment due to evidence of benefits for communication, satisfaction of care, treatment adherence, symptom control, HRQOL, hospital admissions and visits, and survival [6,7]. ePROs are being increasingly integrated as part of routine oncology clinical care in the United Kingdom, the United States, and Australia [8-11]. One study exploring the use of ePROs in clinical practice has shown that symptom and quality-of-life scores reported by patients are clinically meaningful and relevant: cough and mobility scores were lower for people with poorer performance status, and patients undergoing chemotherapy and radiotherapy showed improvements in some symptoms [12].

Previous reviews have examined the features of ePRO systems (eg, exploring the integration of ePROs into clinical practice and identifying features that may be associated with patient engagement and patient-centered outcomes [13,14]). Reviews have also been conducted to examine the impact of ePROs on quality of patient care [15].

### Objectives

To our knowledge, no existing reviews have grouped randomized controlled trials (RCTs) to estimate an effect size through a meta-analysis to establish clinical benefit. Some meta-analyses evaluated the capacity of telehealth or eHealth interventions to enhance HRQOL in patients with cancer [16,17]. However, many of these studies included complex interventions with various components, not just the ePRO alone. Similarly, other reviews have not specifically explored interventions that provide results to clinicians, and they have not explored the specific components of the ePRO interventions [18,19]. The primary objective of our review was to examine the effect of administering ePROs to patients with cancer on HRQOL compared to usual care. The secondary objectives included the comparison of survival, symptoms, psychological well-being, health care use, and satisfaction with care between participants receiving the ePRO intervention and those receiving usual care.

## Methods

### Search Strategy

The review protocol was registered with PROSPERO (CRD42020175007). Subsequent protocol changes included the requirement that ePRO results be fed back to clinicians for review. The reporting of this review was guided by the standards of the PRISMA (Preferred Reporting Items for Systematic Reviews and Meta-Analyses) statement. We used the PRISMA guidelines [20] to ensure that all relevant information detailing the processes we followed as well as the findings of this review were included within the manuscript. The completed PRISMA checklist can be found in Multimedia Appendix 1 [20]. Seven databases (PubMed, MEDLINE, PsycInfo, Cochrane Central Register of Controlled Trials, Web of Science, Embase, and CINAHL) were searched systematically on July 16, 2020 (search 1), and June 20, 2022 (search 2). Similar keywords were used across the databases, adapting Boolean operators and Medical Subject Headings vocabulary. The search terms related to the electronic dimension of the “ePRO” system, “patient reported outcomes,” “cancer,” and “randomized controlled trials.”

### Inclusion Criteria

Full-text research papers in English published in peer-reviewed journals from January 2000 to June 2022 were included in the review. Given the technological advances in this field over recent years, studies published before 2000 were excluded because the ePRO systems they described would not be comparable to currently available ePRO systems. The inclusion criteria are listed in Textbox 1.

**Inclusion criteria.**

**Inclusion criteria and determinants**

- Population: those with any type of cancer diagnosis or cancer stage, aged ≥16 years
- Intervention: electronic patient-reported outcome interventions where participants report outcomes electronically (web-based, computer, mobile phone, tablet, etc), and responses are subsequently made available to clinical teams
- Comparison: usual care or other control conditions
- Outcomes: validated questionnaires measuring health-related quality of life (primary outcome), symptoms, psychological well-being, satisfaction with care, health care use, survival, and progression-free survival (secondary outcomes)

Note: The PICO (Population, Intervention, Comparison, Outcomes) framework to identify determinants [21] was used for the inclusion criteria.

### Exclusion Criteria

We excluded studies based on the criteria presented in Textbox 2.

**The criteria used to exclude studies.**

**Exclusion criteria**

- Book chapters, conference abstracts, commentaries, opinion articles, reviews, meta-analyses, unpublished data, and so on
- Not an electronic patient-reported outcome (ePRO) intervention
- Not focusing on patients with cancer and patients aged ≥16 years
- Not a randomized controlled trial (eg, nonrandomized trial, correlational study, or case study)
- Conditions differ except for the ePRO intervention (eg, the ePRO group received another intervention that was not received by the control group)
- Data published elsewhere
- No control group or an inappropriate control group (eg, where the control condition received an ePRO intervention or another intervention not received by the experimental group)
- Not a health-related outcome measured using validated questionnaires: no health-related quality of life, physical symptoms (eg, nausea, vomiting, pain, breathlessness, and fatigue), psychological symptoms (eg, anxiety and depression), satisfaction with care, health care use outcomes (hospital admission, emergency department visit, and chemotherapy completion), survival, or progression-free survival
- ePRO results were not fed back to clinicians
- Full text not available

### Screening

The identified papers were collated and duplicates removed. The screening of titles and abstracts was conducted independently by 2 reviewers (BK and MBP). Full texts were located for any papers meeting the inclusion criteria and again reviewed by 2 reviewers (BK and MBP). Authors were contacted if full texts could not be obtained. A third reviewer (ST) was consulted in case of any disagreements. Backward and forward reference searching was used to identify additional papers.

### Data Extraction

Data were extracted and recorded by 2 researchers (BK and MBP) and included sociodemographic and clinical information, type of ePRO system, feature included in the ePRO (according to the taxonomy of system features [14]), study design and characteristics, type and validity of outcome measure, dropouts, sample sizes, and data used to compute the effect size.

### Risk-of-Bias Assessment

The risk of bias was assessed by 3 researchers independently (BK, MBP, and AV) according to the revised Cochrane risk-of-bias tool for randomized trials [22] before making a final collaborative decision. Studies were categorized as *low risk of bias*, *some concern*, or *high risk of bias*.

### Data Analysis

The outcomes used and the time points of assessments across the studies were assessed for consistency. If a sufficient number of studies reported common outcomes but measured them in different ways, data were combined as standardized mean differences (SMDs). This is a commonly used summary statistic in meta-analysis that expresses the magnitude of the effect in each study compared to the variability observed. It is calculated by taking the difference in mean outcomes between the groups and dividing it by the SD of the outcome among participants [23]. Where outcomes used the same measurement scale, we combined data as the mean difference. We completed 2 separate meta-analyses: one looking at any HRQOL measure closest to 6 months and the other examining Functional Assessment of Cancer Therapy–General (FACT-G) scores at 3 months; we looked at this particular measure and time point specifically because it was the most commonly used. For all analyses, a maximum likelihood random effects meta-analysis was performed.

The *I*^2^ statistic was used to assess the presence of heterogeneity. There was no observed heterogeneity (*I*^2^=0%), but the 95% CIs were wide (95% CI 0%-68% and 95% CI 0%-79%), suggesting that the *true* heterogeneity could plausibly be high; therefore, random effects were chosen. The causes of heterogeneity were not explored, although, as sensitivity analyses, we repeated the meta-analyses with fixed effects. Forest plots were used to visually present the results. Stata (version 14; StataCorp LLC) was used for all analyses, specifically the *metaan* command [18].

## Results

### Results of the Search

**Figure 1:**
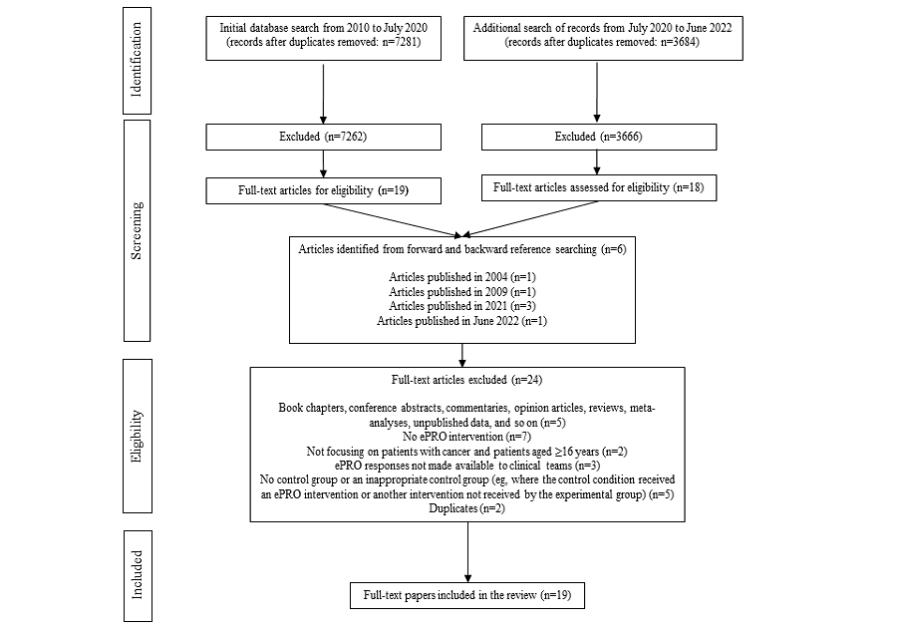
Illustration of the flow of studies through the systematic review process. ePRO: electronic patient-reported outcome.

The search was conducted in 2 stages. A total of 10,965 papers were identified across the 2 searches (2020 and 2022; search 1: n=7281, 66.4%; search 2: n=3684, 33.6%). The full texts of 37 papers were reviewed (search 1: n=19, 51%; search 2: n=18, 49%). Of these 37 papers, 13 (35%) met the inclusion criteria and were included in the review (Figure 1). References from the included papers were reviewed, and this led to 6 additional articles being identified (search 1: n=5, 83%; search 2: n=1, 17%). Thus, overall, 19 papers were deemed eligible after full-text review.

### Included Studies

Nineteen papers from 15 RCTs were identified. The second paper of a study was only used if it provided some additional data not presented in the first paper. Reasons for exclusion are summarized in the flow diagram (Figure 1). The characteristics of the 19 included papers are presented in Table 1, and a summary of the studies is presented in Textbox 3. All patients received systemic anticancer therapy. Each study included in the review compared patient reporting of symptoms to the clinical team via an ePRO to a control group. The majority of the studies (14/15, 93%) compared just 2 groups; however, 1 (7%) of the 15 studies included a third *attention-control* group where participants completed the intervention, but the results were not fed back to the clinical team [24]. The control comparison group was defined in the majority of papers as *standard* or *usual* care. Standard or usual care generally consisted of regular appointments with oncology specialists to assess symptoms, followed by appropriate management. Patients were also encouraged to contact the clinical team by telephone if they experienced any problems between appointments. Of the 15 studies, 2 (13%) [25,26] did not provide a definition of standard or usual care.

**Table 1:** The included papers categorized by study (year and country), methods, sample size, and participant characteristics.

Study, year; country	Methods	Sample size	Participant characteristics
Absolom et al [27], 2021; United Kingdom	RCT^a^; 2 groups; random permuted blocks via an automated 24-hour system	Eligible=782; randomized=508; I^b^=256, C^c^=252; DO: I=42, C=19	Age (y) mean: I=55.9 (SD 12.2), C=56 (SD 11.3) Sex: M^d^=102, F^e^=406 Diagnosis and stage of disease: colorectal, breast, or gynecologic cancers; cancer of any stage
Basch et al [28], 2016; United States	RCT; 2 groups each, with 2 subgroups based on level of prior computer use; computer system using randomly permuted blocks	Eligible=1107; randomized=766; CE^f^ participants: I=286, C=253; CI^g^ participants: I=155, C=72; DO: I=69, C=89 (CE participants); I=46, C=26 (CI participants)	Age (y), median: I=61 (IQR 30-91), C=62 (26-88) Sex: M=322, F=444 Diagnosis and stage of disease: breast, genitourinary, gynecologic, or lung cancers; metastatic
Basch et al [29], 2017; United States	RCT; 2 groups each, with 2 subgroups based on level of prior computer use; computer system using randomly permuted blocks	Eligible: not stated; randomized=766; I=539, C=227; DO (overall): 249	Age (y; overall), median: 61 (IQR 26-91) Sex: M=322, F=444 Diagnosis and stage of disease: breast, genitourinary, gynecologic, or lung cancers; metastatic
Basch et al [30], 2022; United States	RCT; 2 groups; cluster design	Eligible=1444; randomized=1191; I=593, C=598; DO: I=278, C=230	Age (y), median: I=64 (IQR 29-89), C=62 (IQR 28-93) Sex: M=496, F=694; 1197 randomized and 1191 analyzed; however, data on sex are only listed for 1190 patients Diagnosis and stage of disease: cancer of any type; metastatic
Denis et al [31], 2017; France	RCT; 2 groups; minimization program	Eligible: not stated; randomized=133; I=67, C=66; DO: I=7, C=5	Age (y), median: I=65.2 (IQR 35.7-86.9), C=64.3 (IQR 42.7-88.1) Sex: M=81, F=40; 133 randomized and 121 analyzed Diagnosis and stage of disease: nonprogressive small cell lung cancer or non–small cell lung cancer; advanced (at least cTxN1 or pTxpN1 to TxNxM+ at least stage II cancer)
Greer et al [32], 2020; United States	RCT; 2 groups; computer-generated randomization scheme stratified by cancer type	Eligible=500; randomized=181; I=91, C=90; DO: I=11, C=1	Age (y), mean: I=52.85 (SD 13.74), C=53.76 (SD 12.08) Sex: M=84, F=97 Diagnosis and stage of disease: diverse malignancies; not stated
Kearney et al [33], 2009; United Kingdom	RCT; 2 groups; automated interactive voice response telephone randomization system	Eligible: not available due to incomplete data; randomized=112; I=56, C=56; DO: I=27, C=27	Age (y), mean: I=55.1 (SD 10.6), C=56.9 (SD 10.5) Sex: M=26, F=86 Diagnosis and stage of disease: breast, lung, or colorectal cancer; irrespective of stage
Maguire et al [34], 2021; Austria, Greece, Ireland, Norway, and the United Kingdom	RCT; 2 groups; repeated measures parallel group evaluator-masked stratified trial	Eligible=1222; randomized=840; I=422, C=418; DO: I=134, C=122	Age (y), mean: I=51.9 (SD 12.4), C=52.9 (SD 12.1) Sex: M=151, F=678; 840 randomized and 829 analyzable Diagnosis and stage of disease: breast cancer, colorectal cancer, Hodgkin disease, or non-Hodgkin lymphoma; stages 0 to IV (not metastatic breast or colorectal cancer)
Pappot et al [25], 2021; Denmark	RCT; 2 groups; cluster randomization	Eligible=682; randomized=682; I=347, C=335; DO: I=11, C=22	Age (y), median: I=53 and C=53 (range 21-82) Sex: M=0, F=682 Diagnosis and stage of disease: breast cancer; not stated
Post et al [26], 2013; United States	RCT; 2 groups; method of randomization not described	Eligible=93; randomized=60; I=31, C=29; DO: I=4, C=6	Age (y), mean: I=49.5 (SD 10.7), C=52.1 (SD 8.5) Sex: M=0, F=60 Diagnosis and stage of disease: breast cancer; primary (stages I-III)
Riis et al [35], 2020; Denmark	RCT; 2 groups; computer-generated sequence	Eligible=177; randomized=134; I=65, C=69; DO: I=5, C=7	Age (y), mean: I=64.4, C=64.2 Sex: M=0, F=129 Diagnosis and stage of disease: breast cancer; primary (early breast cancer, stages I-III)
Riis et al [36], 2021; Denmark	RCT; 2 groups; computer-generated sequence	Eligible=177; randomized=134; I=65, C=69; DO: I=5, C=7	Age (y; overall), mean: 64.3 Sex: M=0, F=129 Diagnosis and stage of disease: breast cancer; primary (early breast cancer, stages I-III)
Tolstrup et al [37,38], 2020, 2022; Denmark	RCT; 2 groups; open-label, computer-randomized trial	Eligible=200; randomized=146; I=73, C=73; DO: I=6, C=2	Age (y), median: I=66, C=66 Sex: M=78, F=68 Diagnosis and stage of disease: melanoma; metastatic (stages III-IV)
Velikova et al [24], 2004; United Kingdom	RCT; 3 groups; random permuted blocks by telephone	Eligible=439; randomized: 286; I=144, attention-C=70, C=72; DO: I=60, attention-C=35, C=27	Age (y), mean: I=55.1 SD (13.02), attention-C=54.8 (SD 12.4), C=54.7 (SD 11.67) Sex: M=76, F=210 Diagnosis and stage of disease: cancer of any type; mixed stage
Velikova et al [39], 2010; United Kingdom	RCT; 3 groups; telephone by the research office	Eligible=439; randomized=286; I=144; attention-C=70, C=72; DO: I=59, attention-C=36, C=25	Age (y), mean: I=54.8 (SD 12.9), attention-C=55.2 (SD 11.79), C=54.9 (SD 11.76) Sex: M=64, F=194 Diagnosis and stage of disease: cancer of any type; mixed stage
Wheelock et al [40], 2015; United States	RCT; 2 groups; block design	Eligible=102; randomized=100; I=59, C=41; DO: I=9, C=6	Age (y), mean: I=54.78 (SD 8.66), C=3.3 (SD 10.79) Sex: M=0, F=100 Diagnosis and stage of disease: breast cancer; primary (stages I-III)
Yang et al [41], 2019; China	RCT; 2 groups; scheme generated by independent statistical personnel using a computer	Eligible=58; randomized=58; I=31, C=27; DO: 0	Age (y), mean: I=51.1 (SD 8.98), C=53.96 (SD 8.58) Sex: M=38, F=20 Diagnosis and stage of disease: cancer of any type; not stated
Zhang et al [42], 2022; China	RCT; 2 groups; open-label trial	Eligible=364; randomized=300; I=150, C=150; DO: I=9, C=13	Age (y), mean: I=57.6 (SD 12.6), C=60.1 (SD 12.7) Sex: M=206, F=72 Diagnosis and stage of disease: cancer of any type; life expectancy was at least 6 months; not stated

^a^RCT: randomized controlled trial.

^b^I: intervention.

^c^C: control.

^d^M: male.

^e^F: female.

^f^CE: computer-experienced.

^g^CI: computer-inexperienced.

**Summary of included studies.**

**Studies summarized**

- Countries: United States (5/15, 33%), United Kingdom (3/15, 20%), Denmark (3/15, 20%), China (2/15, 13%), France (1/15, 7%), and multiple European countries (1/15, 7%)
- Randomized controlled trials: 2 groups (14/15, 93%) and 3 groups (1/15, 7%)
- Study size: ranging from 58 to 1191 patients, with a total of 5446 patients; 5497 patients randomized
- Sex: 83.6% (4553/5446) were female
- Stage of disease: primary cancer (3/15, 20%), metastatic cancer (4/15, 27%), and any stage or not specified (8/15, 53%)

### Study Quality

The risk-of-bias assessment for each study is summarized in Table 2.

**Table 2:** Results from the risk-of-bias assessment performed using the revised Cochrane risk-of-bias tool for randomized trials.

Study, year	Randomization and allocation (selection bias)	Blinding of participants (performance bias)	Missing outcome data (attrition bias)	Blinding of outcome assessment (detection bias)	Selective reporting (reporting bias)
Absolom et al [27], 2021	Low risk of bias	Low risk of bias	Low risk of bias	Some concerns (outcome assessors were aware of the intervention received by study participants)	Low risk of bias
Basch et al [28,29], 2016 and 2017	Low risk of bias	Low risk of bias	Low risk of bias	Some concerns (outcome assessors were aware of the intervention received by study participants)	Low risk of bias
Basch et al [30], 2022	Low risk of bias	Low risk of bias	Low risk of bias	Some concerns (outcome assessors were aware of the intervention received by study participants)	Low risk of bias
Denis et al [31], 2017	Low risk of bias	Low risk of bias	Low risk of bias	Some concerns (outcome assessors were aware of the intervention received by study participants)	Low risk of bias
Greer et al [32], 2020	Low risk of bias	Low risk of bias	Low risk of bias	Some concerns (outcome assessors were aware of the intervention received by study participants)	Low risk of bias
Kearney et al [33], 2009	Low risk of bias	Low risk of bias	Low risk of bias	Some concerns (outcome assessors were aware of the intervention received by study participants)	Low risk of bias
Maguire et al [34], 2021	Low risk of bias	Low risk of bias	Low risk of bias	Low risk of bias	Low risk of bias
Pappot et al [25], 2021	Low risk of bias	Low risk of bias	Low risk of bias	Some concerns (outcome assessors were aware of the intervention received by study participants)	Low risk of bias
Post et al [26], 2013	Low risk of bias	Low risk of bias	Low risk of bias	Some concerns (outcome assessors were aware of the intervention received by study participants)	Some concerns (did not include a prespecified analysis plan)
Riis et al [35,36], 2020 and 2021	Low risk of bias	Low risk of bias	Low risk of bias	Some concerns (outcome assessors were aware of the intervention received by study participants)	Low risk of bias
Tolstrup et al [37,38], 2020 and 2022	Low risk of bias	Low risk of bias	Low risk of bias	Some concerns (outcome assessors were aware of the intervention received by study participants)	Low risk of bias
Velikova et al [24,39], 2004 and 2010	Low risk of bias	Low risk of bias	Low risk of bias	Some concerns (outcome assessors were aware of the intervention received by study participants)	Low risk of bias
Wheelock et al [40], 2015	Low risk of bias	Low risk of bias	Low risk of bias	Some concerns (outcome assessors were aware of the intervention received by study participants)	Some concerns (provided minimal details of a prespecified analysis plan)
Yang et al [41], 2019	Low risk of bias	Low risk of bias	Low risk of bias	Low risk of bias	Low risk of bias
Zhang et al [42], 2022	Low risk of bias	Low risk of bias	Low risk of bias	Some concerns (outcome assessors were aware of the intervention received by study participants)	Low risk of bias

### ePRO Intervention Components

#### Overview

Table 3 describes the components of the included interventions.

**Table 3:** Intervention components and the effect on review primary outcome and secondary outcomes.

Study, year	Intervention components						Review primary outcome, secondary outcomes, and effect						
	Symptom monitoring	Symptom management	Communication	Alert management	Timing of alerts	Quality of life (primary outcome)		Patient survival	Symptoms	Hospital admissions	Emergency department visits	Chemotherapy completion	Satisfaction with care
Absolom et al [27], 2021	Patients and clinicians (linked to electronic patient records)	Tailored advice for patients; reports sent to clinicians	NR^a^	Clinical team shared email address; monitored by nurses	Real time	+^b^		—^c^	+	–^d^	—	–	—
Basch et al [28,29], 2016 and 2017	Patients and clinicians	Reports sent to clinicians	NR	Email sent to nurses; not monitored 24 hours	Printed at each clinic visit	+		+	+	–	+	+	—
Basch et al [30], 2022	Patients and clinicians	Tailored advice for patients; reports sent to clinicians	NR	Email sent to designated admin staff who forwarded it to relevant nurse	Real time; reports at clinic visits	+		—	+	—	—	—	—
Denis et al [31], 2017	Patients and clinicians	Reports sent to clinicians	NR	Email sent to oncologist	Real time	+		+	—	—	—	—	—
Greer et al [32], 2020	Patients and clinicians	Generic advice for patients; reports sent to clinicians	NR	Email sent to clinician	Not specified	–		—	–	–	–	—	–
Kearney et al [33], 2009	Patients and clinicians	Tailored advice for patients; reports sent to clinicians	NR	Dedicated 24-hour pager system; clinicians should contact patients within 1 hour for severe symptoms	Real time	—		—	+	—	—	—	—
Maguire et al [34], 2021	Patients and clinicians	Tailored advice for patients; reports sent to clinicians	NR	Alerts sent to clinicians on dedicated handsets	Real time	+		—	+	—	—	—	—
Pappot et al [25], 2021	Patients and clinicians	NR	NR	NR	Shown to clinicians after completion at each visit	–		—	–	–	—	–	—
Post et al [26], 2013	Patients and clinicians	Tailored advice for patients	NR	NR	Printed at each clinic visit	–		—	–	—	—	—	—
Riis et al [35,36], 2020 and 2021	Patients and clinicians	Reports sent to clinicians	The patient could request a consultation through the ePROM^e^ system	Principal investigator monitored questionnaire and emails	Not specified	–		—	–	—	—	–	–
Tolstrup et al [37,38], 2020 and 2022	Patients and clinicians	Patients advised to contact clinical team for severe symptoms	NR	NR	Log in to system to view before consultation	+		—	–	—	—	—	—
Velikova et al [24,39], 2004 and 2010	Patients and clinicians	NR	NR	NR	Printed at each clinic visit	+		—	+	—	—	—	–
Wheelock et al [40], 2015	Patients and clinicians	Tailored advice for patients; reports sent to clinicians	Free text to report concerns and ask questions	Monitored by designated nurse practitioner	Real time	—		—	–	—	—	—	—
Yang et al [41], 2019	Patients and clinicians	Patients advised to follow medication plan in case of severe pain	Real-time consultation	NR	Viewed when patients request a consultation	+		—	+	—	—	—	—
Zhang et al [42], 2022	Patients and clinicians	Tailored advice for patients; reports sent to clinical team	Consult team via app at any time	An oncology specialist and 2 nurses from each center	Viewed before consultation	+		–	+	—	+	–	—

^a^NR: not reported.

^b^Statistically significant effect (*P*<.05).

^c^Did not measure this outcome.

^d^No statistically significant effect (*P*>.05).

^e^ePROM: electronic patient-reported outcome measure.

#### Symptom Monitoring

All identified ePROs required patients to monitor and report symptoms and included the facility for a clinician to view results. The majority of the ePROs (14/15, 93%) collected data on a wide range of symptoms, whereas 7% (1/15) focused specifically on pain [41].

#### Symptom Management

Although all ePROs gave the clinical team access to patient reports, 7 (37%) of the 19 studies [24-26,37-39,41] did not actively send reports to the clinical team. Of the 14 systems, 9 (64%) incorporated a facility to alert clinicians if patients reported severe symptoms or a change in symptoms over time [27-31,33-36,40,42]. Greer et al [32] sent all reports (not just those reporting severe symptoms) to the clinical team. Of the 15 interventions, 9 (60%) provided tailored self-management advice for patients based on the problems reported and their severity [26,27,30,33,34,40,42]. In some instances (2/15, 13%), the advice was to contact the clinical team [28,29,40], whereas others (5/15, 33%) provided links to self-management techniques and advice [26,30,33,34,41]. Some (2/15, 13%) used algorithms based on symptom severity to indicate whether patients should receive self-management advice or be advised to contact the clinical team [27,32].

#### Communication

Of the 15 ePROs, 4 (27%) facilitated patient communication with the clinical team [35,36,40-42]. Communication facilities included the ability to contact the team at any time through the app, initiate or request the need for a consultation, and use free text to report concerns and ask questions.

#### Alert Management

The majority of the ePROs (6/15, 40%) sent alerts by email; however, in 2 (13%) of the 15 studies, dedicated handsets or pager systems were used [33,34]. Only 1 (7%) of the 15 studies specified 24-hour alert monitoring [33]. Reports were usually sent to designated clinicians; however, in 1 (7%) of the 15 studies, reports were sent to an administrative team who then directed them to an appropriate member of the nursing team [30]. ePRO questionnaire responses were only integrated into electronic patient record systems in 1 (7%) of the 15 studies [27]. The remaining studies (2/15, 13%) used stand-alone web-based systems that required the clinical team to log in to view ePRO responses.

#### Timing of Alerts

The timing of delivery of ePRO reports to clinicians varied: in 6 (40%) of the 15 studies, electronic reports or alerts were provided in real time [27,30,31,33,34,40]; in 6 (40%) of the 15 studies, reports were reviewed before consultations [24-26,28,29,37-39,42]; and in other studies, reports were reviewed weekly (1/15, 7%) [32], only if patients requested a consultation (1/15, 7%) [41], or if no timing was specified (2/15, 13%) [32,35,36].

### Delivery of the Intervention

The frequency of expected ePRO completion varied across the studies. Some of the studies requested reports at specified time intervals: daily (1/15, 7%) [41], weekly (7/15, 47%) [26,27,30-32,37,38,42], or every third month (2/15, 13%) [35,36,40]. Other studies based ePRO completion around clinical time points: before each clinic visit (2/15, 13%) [24,28,29,39], before each cycle of chemotherapy (2/15, 13%) [25,34], or on days 1 to 14 of each chemotherapy cycle (2/15, 13%) [33]. None of the included studies provided data detailing the fidelity of intervention delivery. Of the 15 studies, 2 (13%) mentioned administrative errors where patient data were not collected due to questionnaires not being given, but this referred to outcome data only and not to the ePRO intervention [32,38].

### Patient Adherence to Allocated Intervention

Data on patient adherence to the trial interventions were available in 10 (67%) of the 15 studies [24-30,33-40]. Patient adherence was not standardized across the studies; rather, it was assessed and reported in various ways. Of the 19 papers, 4 (21%) [25,30,34,40] reported the percentage of intervention completions versus expected intervention completions across the whole study; 4 (21%) [27,33,35,36] reported completion rates by time point, either for individual patients or as an average; 4 (21%) [28,29,37,38] reported the percentage of patients across the whole study who completed the intervention as per protocol; and 1 (5%) [26] reported the percentage of participants who completed reports. Each paper reported an individual adherence rate, and the figures reported were between 50% and 100%, with only 11% (2/19) reporting figures <70% [27,40]. None of the studies reported adherence in terms of whether the interventions were fully or partially completed. Of the 4 papers that presented adherence by time point, Kearney et al [33] and Absolom et al [27] reported a decrease over time (from 100% to 73% and from 72% to 58%, respectively), whereas Riis et al [35,36] reported no significant change over time (*P*=.37).

### Primary Outcome: Quality of Life

Table 4 highlights the intervention focus for the included papers along with the primary outcome and secondary outcomes.

**Table 4:** Intervention types along with the study primary outcome and secondary outcomes.

Study, year	Intervention type	Study primary outcome	Study secondary outcomes
Absolom et al [27], 2021	eRAPID^a^, an online eHealth system for patients to self-report symptoms	Symptom control	Impacts on hospital services (process of care measures) and cost-effectiveness
Basch et al [28,29], 2016 and 2017	Reporting of 12 common symptoms via STAR^b^, a web-based interface	HRQOL^c^	ED^d^ visits, hospitalizations, overall survival, and survival at 1 year
Basch et al [30], 2022	Electronic symptom monitoring with PRO^e^ surveys	Overall survival	Physical function, symptom control, and HRQOL
Denis et al [31], 2017	Web-mediated follow-up of symptoms	Overall survival	Performance status at first relapse, progression-free survival, and HRQOL
Greer et al [32], 2020	Smartphone mobile app	Adherence, symptom burden, and quality of life	Patient satisfaction with treatment and health care use
Kearney et al [33], 2009	Mobile phone–based remote monitoring ASyMS^f^	Symptom scores and the occurrence of 6 symptoms that are components of the total symptom score	—^g^
Maguire et al [34], 2021	Remote monitoring via the ASyMS	Symptom burden	HRQOL, supportive care needs, anxiety, self-efficacy, and work limitations
Pappot et al [25], 2021	ePRO^h^ questionnaire of symptom toxicities	Number of patients with ≥1 treatment adjustments	Number of patients with ≥1 hospitalizations, ≥1 events of febrile neutropenia, number of patients with treatment postponed >7 days, as well as completion of the scheduled 6 cycles of chemotherapy was registered, and compliance to ePRO
Post et al [26], 2013	PDA-delivered symptom communication	Effects on pain, depression, and fatigue symptoms	Study feasibility, patient and clinician responses to study participation, and intervention effects on HRQOL and communication self-efficacy
Riis et al [35,36], 2020 and 2021	A patient-initiated follow-up program customized to the needs of the individual	Satisfaction with care and unmet needs	Use of consultations, adherence to treatment, and quality of life; number of in-person, telephone, and email consultations; and patient satisfaction
Tolstrup et al [37,38], 2020 and 2022	Web-based symptom reporting using AmbuFlex	Number of severe adverse events (grades 3-4)	Service use (eg, number of telephone consultations as well as outpatient and inpatient visits), length of time toxicities experienced, and length of time steroids required; HRQOL; and associations between toxicity severity and HRQOL
Velikova et al [24,39], 2004 and 2010	Touch screen HRQOL questionnaires	HRQOL, physician-patient communication, and clinical management	Process measures (tests, drugs, and medical records), continuity of care, and patient satisfaction
Wheelock et al [40], 2015	An online health questionnaire with a component of remote follow-up called SIS.NET^i^	Quantify the time between symptom reporting and remote evaluation of symptoms	Compare use of health care resources (breast cancer–related visits, total number of medical appointments, and laboratory and imaging studies)
Yang et al [41], 2019	A mobile phone app (Pain Guard)	Remission rate of pain	Medication adherence, improvements in HRQOL, frequency of breakthrough cancer pain, incidence of adverse reactions, and patient satisfaction
Zhang et al [42], 2022	ePRO follow-up mobile app	Incidence of serious (grades 3-4) immune-related adverse events, ED visits, HRQOL, time spent implementing the ePRO model, rate of treatment discontinuation, and death	—

^a^eRAPID: electronic patient self-reporting of adverse events: patient information and advice.

^b^STAR: Symptom Tracking and Reporting.

^c^HRQOL: health-related quality of life.

^d^ED: emergency department.

^e^PRO: patient-reported outcome.

^f^ASyMS: advanced symptom management system.

^g^Not applicable.

^h^ePRO: electronic patient-reported outcome.

^i^SIS.NET: system for individualized survivorship care, based on patient self-reported data, with review by nurse practitioners, targeted education, and triage.

Of the 15 studies, 13 (87%) measured HRQOL, of which 9 (69%) found a statistically significant effect. Only 5 (38%) of these 13 studies [24,26,32,34,40] used a specific tool to measure psychological well-being; in most cases (n=8, 62%), this was measured with a general HRQOL tool. Tolstrup et al [38] found that, at 48 weeks, the intervention group patients had higher mean scores than the control group patients (mean difference 0.06, 95% CI −0.00 to 0.13; *P*=.05). Yang et al [41] found that global HRQOL scores for the ePRO group were significantly higher than those for the control group (*P*<.001). Basch et al [28] found statistically significant improvements for the intervention arm at 6 months for 3 EQ-5D subdomains (mobility: *P*=.02, self-care: *P*=.01, and anxiety and depression: *P*=.01). Improvements in FACT-G scores were reported by Maguire et al [34] (mean difference 4.06, 95% CI 2.65-5.46; *P*<.001) and Velikova et al [24,39] (SE 2.84, 95% CI 13.64-2.37; *P*=.006). Velikova et al [24] found statistically significant changes in FACT-G physical well-being and FACT-G functional well-being subscale scores in particular (*P*=.03). Zhang et al [42] found higher total mean scores for HRQOL in the intervention group at 6 months (mean 74.2, SD 15.1, 95% CI 71.7-76.9 vs mean 64.7, SD 28.5, 95% CI 61.0-68.4; *P*=.01), particularly physical function (mean 84.9, SD 10.5, 95% CI 82.9-88.5 vs mean 68.8, SD 20.7, 95% CI 65.8-72.5; *P*=.001). Basch et al [30] found that patients in the ePRO group had significantly greater HRQOL than those in the usual care group (odds ratio 1.41, 95% CI 1.10-1.81; *P*=.006). Absolom et al [27] found that participants in the intervention group reported better overall health on the EQ-5D visual analog scale at 18 weeks (mean 75.6, SD 18.0 vs mean 68.7, SD 20.4; mean 4.48, 95% CI 1.11-7.86; *P*=.009) and 12 weeks (mean 74.0, SD 16.6 vs mean 71.4, SD 19.5; mean 3.50, 95% CI 0.35-6.66; *P*=.03), but there was no difference at 6 weeks (mean 74.0, SD 17.3 vs mean 71.4, SD 19.5; mean 1.36, 95% CI 21.66-4.39; *P*=.38). Denis et al [31] reported that HRQOL at 6 months was stable or that it improved more in the experimental arm (81% vs 59%; *P*=.04). Of the 15 studies, 4 (27%) [25,26,32,35,36] found no statistically significant differences in relation to HRQOL between groups, and 2 (13%) did not examine HRQOL [28,29,40].

### Meta-Analysis

**Figure 2:**
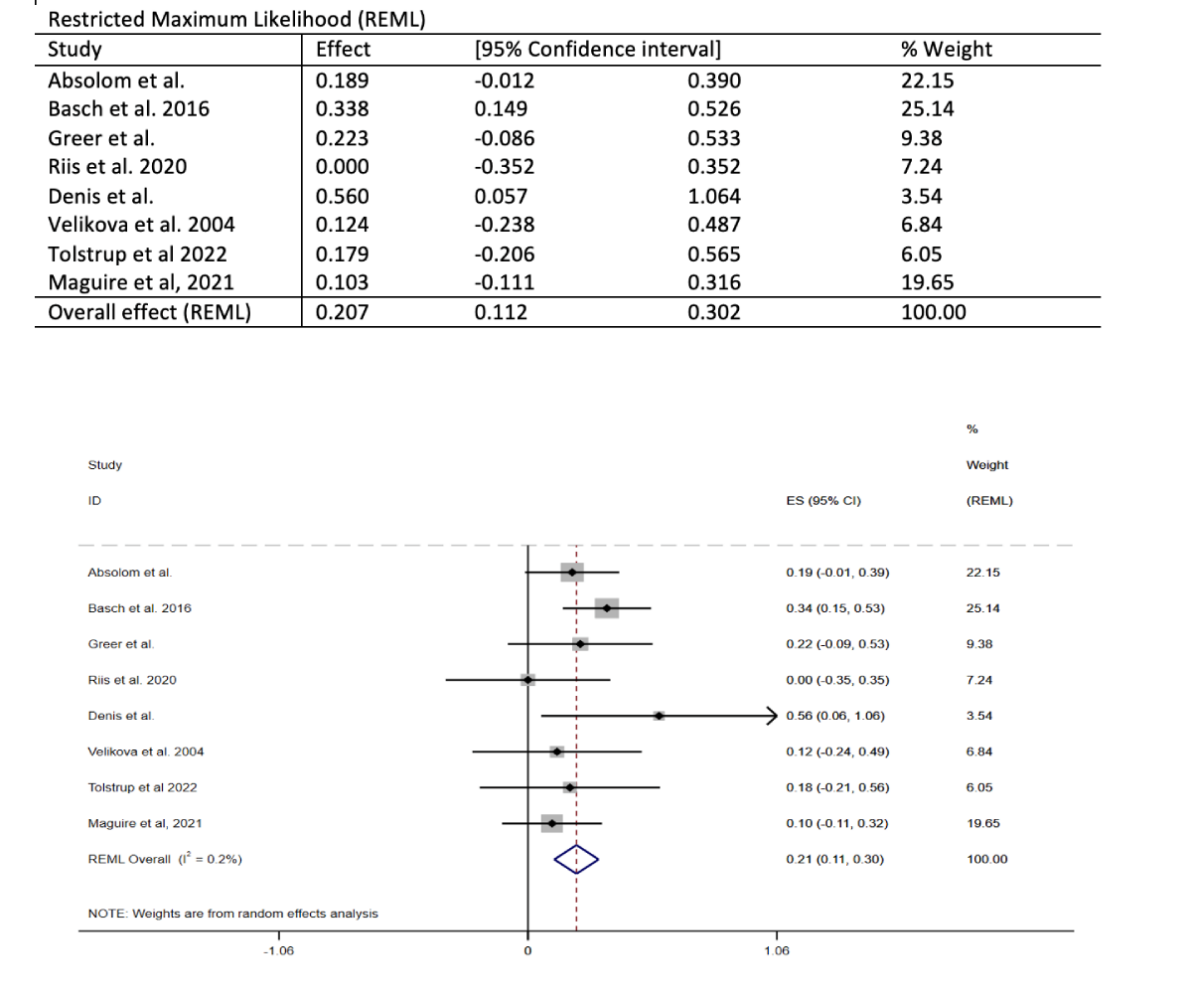
Forest plot of the meta-analysis exploring the effect of any health-related quality of life measure at 6 months.

**Figure 3:**
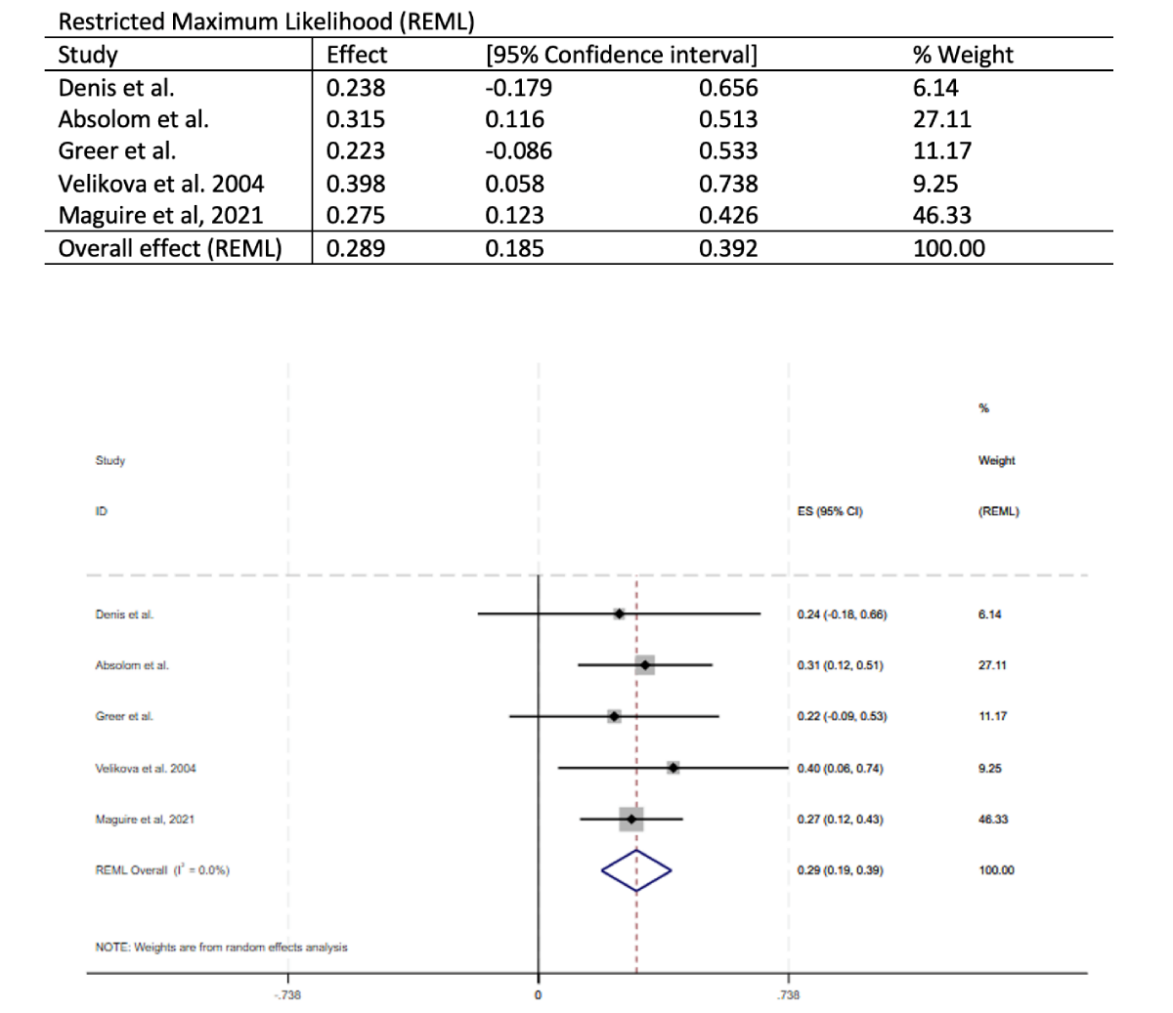
Forest plot of the meta-analysis exploring the effect of treatment on Functional Assessment of Cancer Therapy–General scores at 3 months. ES: effect size.

For RCTs with >1 paper (eg, Basch et al [28,29]), only 1 paper was included in the meta-analysis. Of the 15 studies, 8 (53%) were included in the meta-analysis exploring the effect of any HRQOL measure closest to 6 months (Figure 2 [24,27,28,31,32,34,35,38]). Overall, treatment at 6 months demonstrated an average small improvement (SMD 0.21, 95% CI 0.11 to 0.30). There was relatively little variability in reported effect sizes, which ranged from 0 to 0.56, although the 95% CIs surrounding these values often crossed 0. Of the 15 studies, 5 (33%) were included in a meta-analysis of FACT-G scores at 3 months (Figure 3 [24,27,31,32,34]). Here too, the effect of treatment on FACT-G scores at 3 months showed a small average improvement (SMD 0.29, 95% CI 0.19 to 0.39).

### Secondary Outcomes

#### Patients’ Survival

Of the 19 papers, 4 (21%) reported survival data [28,31,42], with Basch et al [29] performing a follow-up survival analysis. Basch et al [28] found a statistically significant difference in patients’ overall survival at 1 year (75% vs 69%; *P*=.05) and in quality-adjusted survival (mean 8.7 vs 8.0 mo; *P*=.004). Basch et al [29] also explored longer-term survival and reported a significant difference between groups (mean 31.2 months for the ePRO group compared to mean 26.0 months for the usual care group; *P*=.03). Denis et al [31] reported an improvement of 7 months in overall survival for the intervention group (hazard ratio 0.32, 95% CI 0.15 to 0.67; *P*=.002). Zhang et al [42] found no significant differences in survival between the 2 groups (hazard ratio 0.38, 95% CI 0.07-1.99; *P*=.28). The methodologies used by the studies to calculate survival rates were different and included logistic regression [28], the Kaplan-Meier method with log rank tests [29,31], and chi-square tests [42]. Due to the inconsistent methods of comparison, we compared these descriptively rather than in a meta-analysis.

#### Patient Symptoms

Of the 19 papers, 14 (74%) reported the effect of the intervention on patients’ symptoms, as shown in Table 3. Of these 14 papers, 7 (50%) reported a positive effect on symptoms at 5 to 6 months or after 6 cycles of chemotherapy [24,28-30,34,39,42]. Velikova et al [24] found statistically significant changes in FACT-G physical well-being subscale scores in the intervention group compared to the control group (*P*=.006). Maguire et al [34] found that symptoms were better controlled in the intervention group, remaining at pretreatment levels, whereas the symptoms of control group participants increased initially (least squares absolute mean difference –0.15, 95% CI –0.19 to –0.12; *P*<.001).

Other studies reported the impact of the ePRO on symptoms at 3 months. Absolom et al [27] found that FACT-G physical well-being subscale scores showed that there was better symptom control for participants in the electronic patient self-reporting of adverse events: patient information and advice arm at 6 weeks (difference of least squares means 1.08, SE 0.49, 95% CI 0.12 to 2.05; *P*=.03) and 12 weeks (difference of least squares means 1.01, SE 0.49, 95% CI 0.05-1.98; *P*=.04), but there was no difference at 18 weeks (difference of least squares means 0.2, SE 0.51, 95% CI 20.81-1.20; *P*=.70). Basch et al [30] found a significant improvement in symptom control in the intervention group compared to the control group (odds ratio 1.50, 95% CI 1.15-1.95; *P*=.003). Kearney et al [33] found that patients who had received the intervention reported lower levels of fatigue than those in the control group (67% vs 81%; *P*=.04).

Yang et al [41] reported a positive effect on symptoms at 2 weeks and 4 weeks. Yang et al [41] found significant improvements in various symptoms (nausea and vomiting: W=272; *P*=.01; constipation: W=261; *P*=.008; fatigue: W=211.5; *P*=.001; and pain: W=177; *P*<.001) in the intervention group compared to the control group. Of the 19 papers, 6 (32%) reported that the ePRO interventions had no statistically significant effect on symptom control [25,26,32,35,37,40], whereas 1 (5%) paper did not report this outcome [31].

#### Hospital Admissions

Of the 19 papers, 4 (21%) reported the effect of the intervention on hospital admissions. Basch et al [28] reported that the patients in the intervention group were less frequently hospitalized at 1 year (incidence rate: 45% vs 49%; *P*=.08). The remaining papers (3/4, 75%) reported no significant difference in hospital admissions [25,27,32]. All studies assessed the statistical significance differently and at different time points; for example, Absolom et al [27] used incidence rate ratio over 18 weeks, whereas Pappot et al [25] used odds ratio over 6 cycles of treatment.

#### Emergency Department Visits

Of the 19 papers, 3 (16%) reported a positive effect of the ePRO intervention on emergency department (ED) visits. Basch et al [28] found that patients receiving the intervention were less frequently admitted to the ED (34% vs 41%; *P*=.02), as did Zhang et al [42] (23% vs 41%; hazard ratio 0.46, 95% CI 0.26-0.81; *P*=.01). Greer et al [32] reported fewer ED visits resulting in hospitalization (*P*=.05). All studies assessed the statistical significance differently and at varying time points, using incidence at 1 year [28], hazard ratio after the intervention (6 mo) [42], and mean (SE) during the 12-week study period [32].

#### Chemotherapy Completion

Of the 19 papers, 5 (26%) reported the effect of the intervention on chemotherapy completion. Basch et al [28] found that the intervention group remained on chemotherapy longer (mean 8.2 vs 6.3 mo; *P*=.002). Pappot et al [25] found that the 6 scheduled cycles of chemotherapy were completed with treatment adjustments in 34% (ePRO arm) and 40.6% (usual care arm) of the participants (*P*=.10), but this result was not significant. Absolom et al [27], Riis et al [35], and Zhang et al [42] found no significant difference in treatment adherence at the end of the study.

#### Patient Satisfaction With Care

Of the 19 papers, 4 (21%) reported the effect of the intervention on patient satisfaction with care [24,32,35,39]. None of the papers reported a statistically significant difference between the groups. Velikova et al [39] found that between 79% and 89% of the patients, regardless of the study arm, rated their quality of care as *very good*/*excellent*. Similarly, Riis et al [35] found that participants in both groups were highly satisfied with their follow-up care.

## Discussion

### Principal Findings

To our knowledge, this is the first meta-analysis to explore the effects of ePROs on health-related outcomes in adults with cancer. All interventions involved patients reporting their symptoms, which clinicians could view and monitor. The presentation of patients’ ePRO responses to clinicians varied across the studies. Of the 14 systems, only 1 (7%) embedded reports within the electronic patient record system [27]. Some of the studies (6/15, 40%) provided results to clinicians in real time [27,30,31,33,34,40], whereas others (6/15, 40%) distributed results before consultations [24-26,28,29,37-39,42]. Of the 8 systems that included clinician alerts and reported HRQOL, 6 (75%) had a positive effect [27-31,34,42]. Of the 9 systems that alerted clinicians and reported symptoms, 6 (67%) had a positive effect [27-30,33,34,42]. This suggests that alerting clinicians is a key component of ePROs and likely to lead to positive effects on symptoms and HRQOL. None of the studies explored whether the intervention was more effective for particular patient groups, such as a particular sex or cancer type.

The meta-analysis showed an improvement in HRQOL at 6 months. The FACT-G scores at 3 months also showed a small average improvement. Due to the heterogeneity of the studies, specifically the different outcome measures and the different data collection time points, not all studies were included in the meta-analysis. Only 5 (33%) of the 15 studies were included in the FACT-G 3-month meta-analysis, with the weighting predominantly spread across 2 (40%) studies [27,34]. These 2 studies both provided advice for patients and sent reports to clinicians. Of the 15 studies, 8 (53%) were included in the HRQOL 6-month meta-analysis, with the majority of the weighting spread across 3 (38%) studies [27,28,34]. Of the 15 studies, 9 (60%) reported a statistically significant effect on HRQOL. The measures used to assess HRQOL, the specific HRQOL elements that improved, and the time points when significant differences were recorded varied across the studies.

Symptoms were the most commonly reported secondary outcome, and a positive effect was reported in 8 (57%) of the 14 studies where this was measured. The symptoms tracked by ePROs varied across each of the studies however all studies covered common treatment side effects such as fatigue, pain, and nausea. A variety of different tools were used to measure symptoms; therefore, it is difficult for comparisons to be made. Some symptom assessment tools covered a wide range of symptoms, whereas others focused on single symptoms. A narrative review has highlighted the potential benefits of ePRO use in outpatient care in terms of symptom monitoring and facilitating more timely interventions [6]. However, the review does not explore individual components of the interventions, which may significantly impact effectiveness.

Only 3 (20%) of the 15 studies measured survival [28,31,42], of which 2 (67%) reported improved survival in the intervention group [28,31], whereas 1 (33%) found no difference [42]. Many of the other studies only included participants with early disease treated with curative intent; therefore, including survival is not appropriate. Studies exploring the use of ePRO interventions in patients with advanced disease should consider including survival in their outcome measures. A meta-analysis exploring the prognostic values of PROs for survival in cancer suggests that there is evidence of a relationship between PRO data, particularly physical functioning as measured by the European Organisation for Research and Treatment of Cancer Quality of Life Core Questionnaire-30 [43]. Another meta-analysis of 6 studies tentatively suggested that patient monitoring via PROs could have a positive effect on survival [44], but only two-thirds of the studies included ePROs, and only one-third were RCTs. In terms of the impact on care and hospital use, only 2 (13%) of the 15 studies [28,42] reported a significant effect on ED visits. The study by Basch et al [28] also reported a significant impact on hospital admissions and chemotherapy completion. In Basch et al [28,29], patients in the intervention arm remained on chemotherapy for significantly longer. The intervention is similar to other interventions in that symptom reports are available to clinicians, alerts are sent to the nursing team, and results are printed at each clinic visit. The study by Basch et al [28] discusses the direct actions taken after patients report problems. These actions included symptom management counseling, supportive medication changes, referral to the ED or hospital, chemotherapy dose modification, and imaging or test orders. All these actions may have had an impact on chemotherapy tolerance and continued adherence.

Of the 15 studies, 14 (93%) had some methodological concerns, which could indicate a potential risk of bias. However, no study was categorized as *high risk of bias* in any of the categories. Blinding was not possible, given the nature of the intervention.

### Limitations

There are several limitations to this review, which make it difficult to draw firm conclusions regarding the effectiveness of ePRO interventions in clinical practice. In some of the studies (7/15, 47%), the data presented were insufficient for use in the meta-analysis. Authors were contacted where possible, but the required data were not always available. The ability to combine and compare results was further weakened by the varied outcome measures collected at different time points. Many of the studies (12/15, 80%) included a higher proportion of female participants. Male participants may not be as engaged with ePRO interventions or may not openly discuss health problems; therefore, ePRO interventions may not be as effective for male participants.

### Comparison With Prior Work

ePROs have been successfully implemented in clinical practice in other diagnostic groups. In rheumatology, ePROs were found to be feasible for use and provided a unique insight into patient experience [45]. Similarly, in diabetes, the completion of preconsultation ePROs was reported to be feasible and acceptable [46]. The AmbuFlex system was successfully used in follow-up across 9 diagnostic groups, including heart disease, epilepsy, asthma, and some cancers. ePROs were completed and the results used to indicate whether a follow-up appointment was necessary. In line with the studies identified in this review, all these systems included clinician review of symptoms to direct patient management and clinical decisions.

New and more effective oncology treatments are being introduced, meaning that the patient population and clinical workload are increasing. The European Society for Medical Oncology has published guidelines on ePRO use in oncology, providing further evidence on the effectiveness of this approach [7]. Symptom reporting could be useful in helping clinicians to manage a larger number of patients for a longer period of time. The findings from this review can highlight which interventions (and components) may be most useful to support clinical practice and improve patient care. Including a facility to send alerts to clinicians is an important feature that should be included in ePROs aiming to improve HRQOL. Regardless of the features included, any ePRO system will only be a success if patients and clinicians fully engage with it [47]. Patients need to regularly complete PROs, and clinicians need to review results and respond.

### Conclusions

The aim of this systematic review and meta-analysis was to synthesize all current evidence on the effectiveness of ePROs in RCTs. In total, 19 papers of 15 RCTs were identified. Nearly two-thirds (9/15, 60%) of the interventions showed positive effects on HRQOL and symptoms in adults with cancer. However, caution should be taken in interpreting the results of this review due to the heterogeneity in the interventions, outcome measures, and data collection time points. This systematic review should act as a driver for further RCTs to be conducted to explore the effectiveness of ePROs using validated outcome measures. This will help to orientate health care professionals toward the most suitable setting and ePRO features to develop and propose optimal care to patients with cancer.

## Appendix

Multimedia Appendix 1 Completed PRISMA (Preferred Reporting Items for Systematic Reviews and Meta-Analyses) checklist.

## Abbreviations

ED: emergency department
ePRO: electronic patient-reported outcome
FACT-G: Functional Assessment of Cancer Therapy–General
HRQOL: health-related quality of life
PRISMA: Preferred Reporting Items for Systematic Reviews and Meta-Analyses
PRO: patient-reported outcome
RCT: randomized controlled trial
SMD: standardized mean difference

## Data Availability

Data sharing is not applicable to this paper as no data sets were generated or analyzed during this study.
