# Correction: Examining the Effectiveness of Electronic Patient-Reported Outcomes in People With Cancer: Systematic Review and Meta-Analysis

**DOI:** 10.2196/72687

**Published:** 2025-03-11

**Authors:** Melissa Betty Perry, Sally Taylor, Binish Khatoon, Amy Vercell, Corinne Faivre-Finn, Galina Velikova, Antonia Marsden, Calvin Heal, Janelle Yorke

**Affiliations:** 1 Christie Patient Centred Research The Christie NHS Foundation Trust Manchester United Kingdom; 2 Division of Nursing Midwifery and Social Work School of Health Sciences, Faculty of Biology, Medicine & Health The University of Manchester Manchester United Kingdom; 3 Division of Cancer Science The University of Manchester Manchester United Kingdom; 4 Clinical Oncology Department The Christie NHS Foundation Trust Manchester United Kingdom; 5 Leeds Institute of Medical Research University of Leeds Leeds United Kingdom; 6 St James’s Institute of Oncology St James’s University Hospital Leeds United Kingdom; 7 Division of Population Health Health Services Research & Primary Care The University of Manchester Manchester United Kingdom; 8 School of Nursing The Hong Kong Polytechnic University Kowloon China (Hong Kong)

In Examining the Effectiveness of Electronic Patient-Reported Outcomes in People With Cancer: Systematic Review and Meta-Analysis (J Med Internet Res 2024;26:e49089) the authors (notably the study statistician who conducted the analyses, Calvin Heal) noted an error in formulae used to calculate the SE of the standardized mean difference (SMD).

In the Abstract, Results, the following sections:

Results: The search identified 10,965 papers, of which 19 (0.17%) from 15 studies were included. The meta-analysis showed an improvement in HRQOL at 3 months, measured by the Functional Assessment of Cancer Therapy–General (SMD 0.28, 95% CI –1.22 to 1.78), and at 6 months, assessed using various HRQOL measures (SMD 0.07, 95% CI –1.24 to 1.39). The results should be interpreted with caution, given the wide 95% CIs. Of the 15 studies, 9 (60%) reported a positive signal on HRQOL, with two-thirds of the studies (n=6, 67%) including tailored patient advice and two-thirds (n=6, 67%) using clinician alert systems.

Conclusions: The meta-analysis showed a potential improvement in HRQOL at 6 months and in Functional Assessment of Cancer Therapy–General scores at 3 months for studies that included tailored advice and clinician alerts, suggesting that these elements may improve ePRO effectiveness. The findings will provide guidance for future use and help health care professionals choose the most suitable ePRO features for their patients.

Have been revised to:

Results: The search identified 10,965 papers, of which 19 (0.17%) from 15 studies were included. The meta-analysis showed an improvement in HRQOL at 3 months, measured by the Functional Assessment of Cancer Therapy–General (SMD 0.29, 95% CI 0.19 to 0.39), and at 6 months, assessed using various HRQOL measures (SMD 0.21, 95% CI 0.11 to 0.30). Of the 15 studies, 9 (60%) reported a positive signal on HRQOL, with two-thirds of the studies (n=6, 67%) including tailored patient advice and two-thirds (n=6, 67%) using clinician alert systems.

Conclusions: The meta-analysis showed an improvement in HRQOL at 6 months and in Functional Assessment of Cancer Therapy–General scores at 3 months for studies that included tailored advice and clinician alerts, suggesting that these elements may improve ePRO effectiveness. The findings will provide guidance for future use and help health care professionals choose the most suitable ePRO features for their patients.

In the main text, in the Methods section, Data analysis, the following text:

There was no observed heterogeneity (I2=0%), but the 95% CIs were wide (95% CI 0%-64.8% and 95% CI 0%-79.2%), suggesting that the true heterogeneity could plausibly be high; therefore, random effects were chosen.

Has been revised to:

There was no observed heterogeneity (I2=0%), but the 95% CIs were wide (95% CI 0%-68% and 95% CI 0%-79%), suggesting that the true heterogeneity could plausibly be high; therefore, random effects were chosen.

In the Results section, Primary Outcome: Quality of Life, the following text:

Improvements in FACT-G scores were reported by Maguire et al [34] (SMD 4.06, 95% CI 2.65-5.46; *P*<.001) and Velikova et al [24,39] (SE 2.84, 95% CI 13.64-2.37; *P*=.006).

Has been revised to:

Improvements in FACT-G scores were reported by Maguire et al [34] (mean difference 4.06, 95% CI 2.65-5.46; *P*<.001) and Velikova et al [24,39] (SE 2.84, 95% CI 13.64-2.37; *P*=.006).

In the Results section, Meta-Analysis, the following text:

Overall, treatment at 6 months demonstrated an average small improvement (SMD 0.07, 95% CI –1.24 to 1.39), although with a wide 95% CI, suggesting that an effect in either direction is possible. There was relatively little variability in reported effect sizes, which ranged from –0.22 to 0.56, although the 95% CIs surrounding these values were often wide. Of the 15 studies, 5 (33%) were included in a meta-analysis of FACT-G scores at 3 months (Figure 3 [24,27,31,32,34]). Here too, the effect of treatment on FACT-G scores at 3 months showed a small average improvement with a wide 95% CI (SMD 0.28, 95% CI –1.22 to 1.78), again suggesting that the true effect of treatment could be positive, negative, or null.

Has been revised to:

Overall, treatment at 6 months demonstrated an average small improvement (SMD 0.21, 95% CI 0.11 to 0.30). There was relatively little variability in reported effect sizes, which ranged from 0 to 0.56, although the 95% CIs surrounding these values often crossed 0. Of the 15 studies, 5 (33%) were included in a meta-analysis of FACT-G scores at 3 months (Figure 3 [24,27,31,32,34]). Here too, the effect of treatment on FACT-G scores at 3 months showed a small average improvement (SMD 0.29, 95% CI 0.19 to 0.39).

In the Discussion section, the following text:

The meta-analysis showed an improvement in HRQOL at 6 months, although caution should be taken when interpreting the results due to the wide 95% CI. FACT-G scores at 3 months showed a small average improvement but again with a wide 95% CI. Due to the heterogeneity of the studies, specifically the different outcome measures and the different data collection time points, not all studies were included in the meta-analysis. Only 5 (33%) of the 15 studies were included in the FACT-G 3-month meta-analysis, with the weighting predominantly spread across 3 (60%) studies [27,32,34]. These 3 studies all provided advice for patients and sent reports to clinicians. Of the 15 studies, 8 (53%) were included in the HRQOL 6-month meta-analysis, with the majority of the weighting spread across 4 (50%) studies [27-29,32,35,36].

Has been revised to:

The meta-analysis showed an improvement in HRQOL at 6 months. The FACT-G scores at 3 months also showed a small average improvement. Due to the heterogeneity of the studies, specifically the different outcome measures and the different data collection time points, not all studies were included in the meta-analysis. Only 5 (33%) of the 15 studies were included in the FACT-G 3-month meta-analysis, with the weighting predominantly spread across 2 (40%) studies [27,34]. These 2 studies both provided advice for patients and sent reports to clinicians. Of the 15 studies, 8 (53%) were included in the HRQOL 6-month meta-analysis, with the majority of the weighting spread across 3 (38%) studies [27,28,34].

In Conclusions, the following text:

In total, 19 papers of 15 RCTs were identified. Nearly two-thirds (9/15, 67%) of the interventions showed positive effects on HRQOL and symptoms in adults with cancer. However, caution should be taken in interpreting the results of this review due to the heterogeneity in the interventions, outcome measures, and data collection time points, as well as the wide 95% CIs...

Has been revised to:

In total, 19 papers of 15 RCTs were identified. Nearly two-thirds (9/15, 60%) of the interventions showed positive effects on HRQOL and symptoms in adults with cancer. However, caution should be taken in interpreting the results of this review due to the heterogeneity in the interventions, outcome measures, and data collection time points

Also, Figures 2 and 3 have been revised to the following:

**Figure 2:**
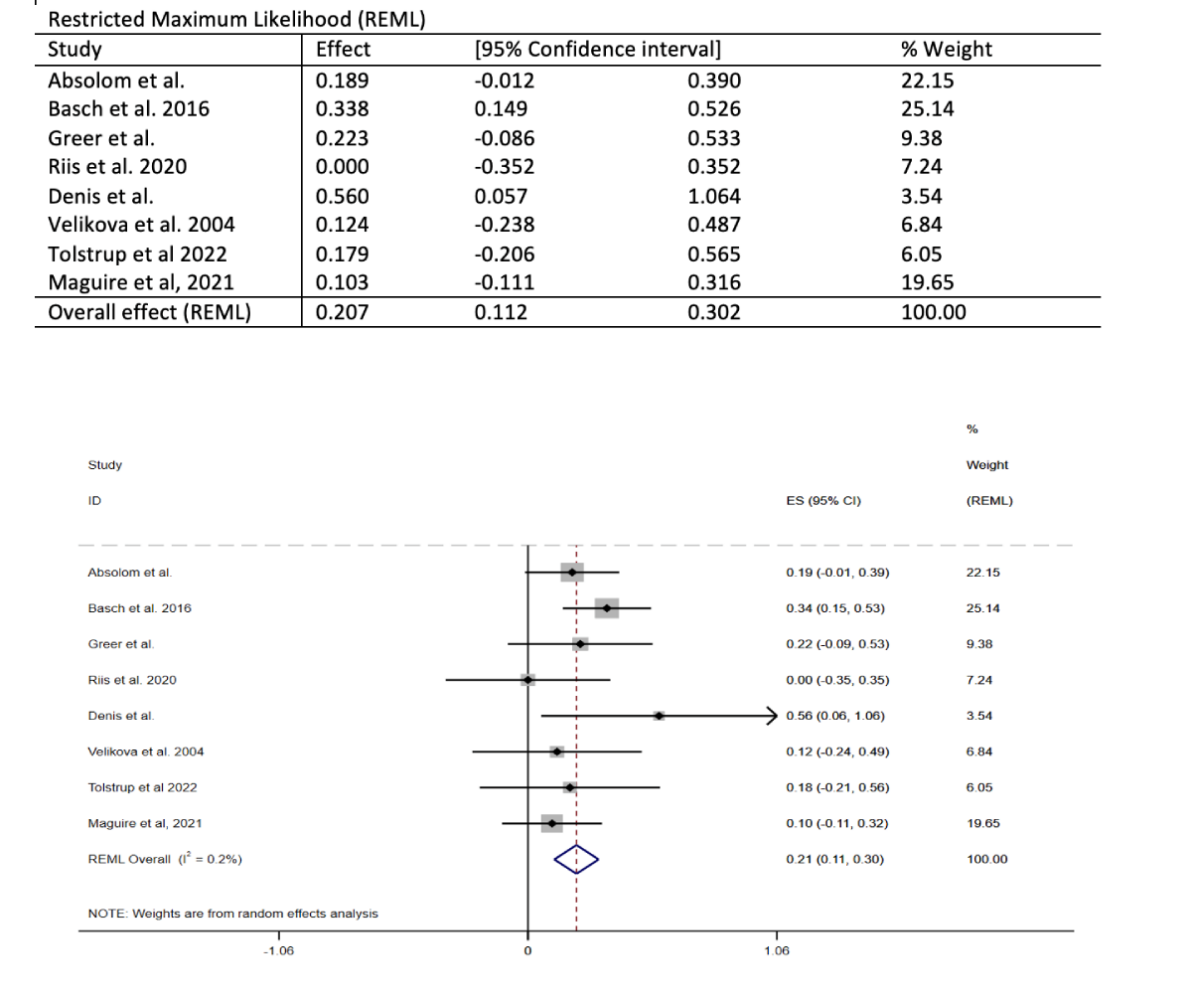
Forest plot of the meta-analysis exploring the effect of the intervention on any health-related quality of life measure at 6 months.

**Figure 3:**
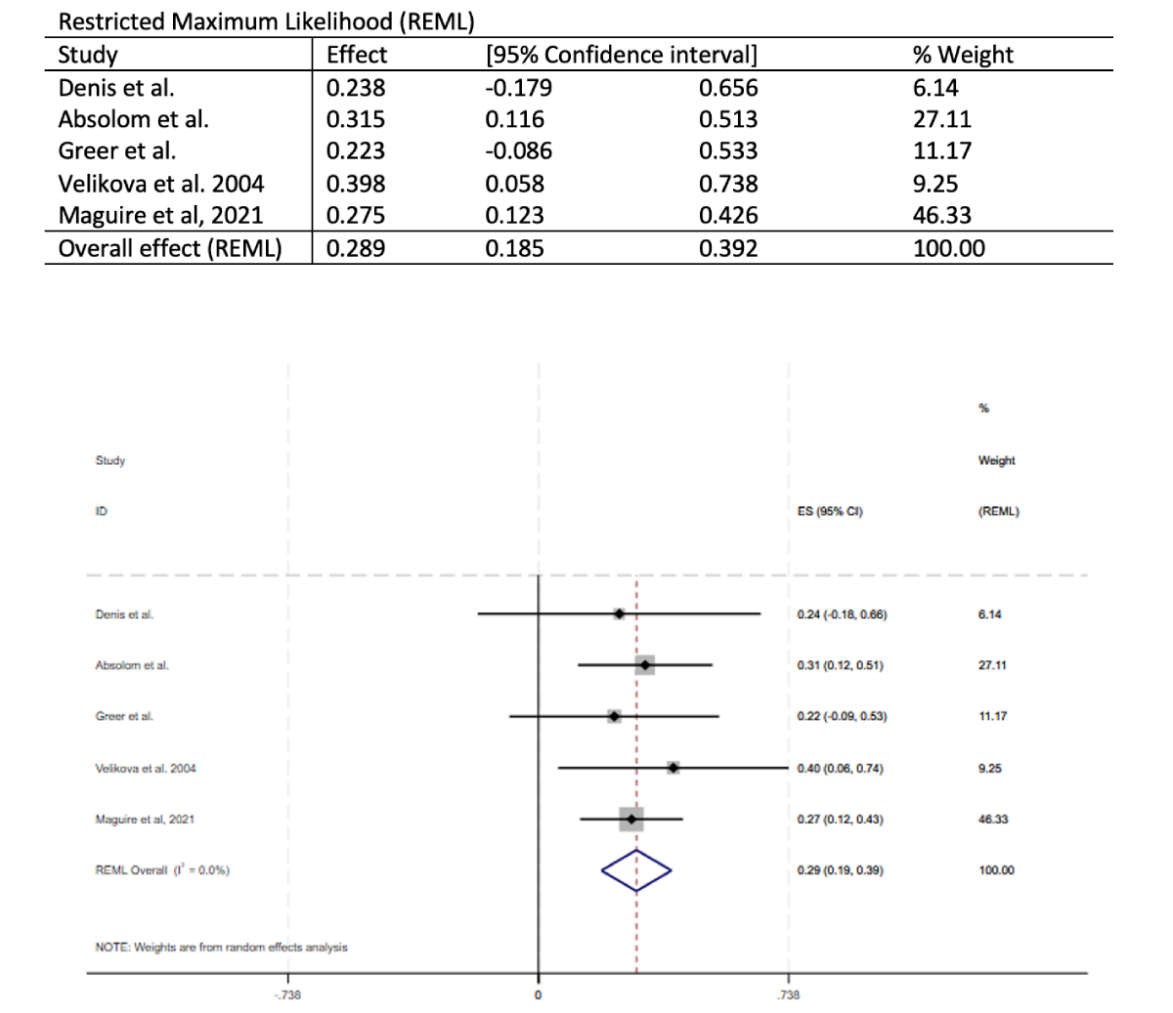
Forest plot of the meta-analysis exploring the effect of treatment on the Functional Assessment of Cancer Treatment-General scores at 3 months.

The correction will appear in the online version of the paper on the JMIR Publications website on March 11, 2025, together with the publication of this correction notice. Because this was made after submission to PubMed, PubMed Central, and other full-text repositories, the corrected article has also been resubmitted to those repositories.

